# The Extent of Tumor in the Peritoneum and Liver Influences Outcomes After Surgery for Synchronous Liver and Peritoneal Colorectal Metastases: A Cohort Study

**DOI:** 10.1007/s12029-024-01139-y

**Published:** 2024-11-14

**Authors:** Pearl Sanchez Salas, Jozef Urdzik, Wilhelm Graf, Bengt Isaksson, Helgi Birgisson

**Affiliations:** 1 Department of Surgical Sciences, Uppsala University, Akademiska Sjukhuset, 751 85 Uppsala, Sweden; 2 Department of Surgery, Orthopedics and Urology, Södertälje Sjukhus, 152 86 Södertälje, Sweden; 3 Department of Surgery, Akademiska Sjukhuset, 751 85 Uppsala, Sweden

**Keywords:** Colorectal cancer, CRS, HIPEC, Liver resection, Peritoneal metastases, Liver metastases

## Abstract

**Purpose:**

Cytoreductive surgery and hyperthermic intraperitoneal chemotherapy (CRSH) or liver resection have led to increased survival in patients with peritoneal or liver metastases of colorectal cancer. Selected patients undergo concomitant CRSH and liver resection. Differences in survival and morbidity between patients who underwent concomitant surgery, CRSH or liver resection for peritoneal and/or liver metastases were compared.

**Methods:**

Patients who underwent liver resection and/or CRSH for colorectal liver and/or peritoneal metastases, 2006–2016, were included. Regression analysis was used to evaluate the associations between baseline characteristics and survival.

**Results:**

Overall, 634 patients were studied. Twenty-eight patients had peritoneal and liver metastases, 121 patients had peritoneal metastases only, and 485 patients had isolated liver metastases. Median survival after concomitant treatment was 23.8 months (95% CI 12.8–43.8), after CRSH 34.5 months (95% CI 27.1–41.9), and after liver resection 54.2 months (95% CI 47.4–61.0) (*p* < 0.001). Increased hepatic tumor burden (HR 3.2, 95% CI 1.8–5.8) and high-volume peritoneal disease (HR 6.0, 95% CI 3.7–9.8) were associated with decreased survival in multivariate analysis. Postoperative complications according to a Clavien–Dindo score > 3a were observed in 11% in the liver resection group, 15% in the CRSH group, and 11% in the concomitant treatment group (*p* = 0.945).

**Conclusions:**

Patients treated with concomitant surgery for liver and peritoneal metastases experienced a shorter median overall survival than patients treated for metastases at an isolated site but had a similar rate of severe postoperative complications. The extent of peritoneal spread seemed to impact survival more than the tumor burden in the liver.

**Supplementary Information:**

The online version contains supplementary material available at 10.1007/s12029-024-01139-y.

## Background

Increased survival has been noted in patients with colorectal cancer peritoneal and liver metastases treated with radical resection, sometimes in combination with chemotherapy [1, 2]. In recent decades, the combination of cytoreductive surgery (CRS) and hyperthermic intraperitoneal chemotherapy (HIPEC) has evolved as a treatment option for selected patients with colorectal cancer and peritoneal metastases [2], and thorough patient selection has led to increased survival [3]. Best practice systemic chemotherapy for colorectal peritoneal metastases has resulted in a median survival of 16.3 months (95% CI 13.5–18.8) and for liver metastases, 19.1 months (95% CI 18.3–19.8) [4]. Van der Geest et al. [5] reported a gradual improvement in median survival, after diagnosis of metastases, over time, e.g., 6.7–9.0 months for peritoneal metastases and 8.8–15.6 months for liver metastases, when comparing the years 1996–1999 and 2008–2011. In a review by Baratti et al. [3], a median survival of 9–32 months for patients with colorectal peritoneal metastases treated with chemotherapy (in the latter case, in combination with cytoreductive surgery) and 16–51 months for patients treated with cytoreductive surgery and HIPEC (CRSH) was reported. For patients having undergone radical resection of colorectal liver metastases with or without neoadjuvant chemotherapy, a review by Kassahun [1] quoted overall survival (OS) ranging from 36 to 65 months, and according to the Swedish population-based liver surgery registry, SWELIV [6], 5-year overall survival after liver surgery for patients with colorectal cancer liver metastases is 45% (2009–2021).

Furthermore, selected patients with colorectal liver and peritoneal metastases undergo concomitant CRS, HIPEC, and liver resection (CRSH + LRx). In a review by Flood et al. [7], 5-year overall survival was 29%, and the OS ranged from 15 to 45 months. A review by Di Carlo et al. [8] reported a mean OS of 30 months.

Although evolving treatments for metastasized colorectal cancer continue to improve patient survival [5], the abovementioned major surgical treatments are taxing for patients as well as costly, and it remains debatable whether or not these combined procedures are medically sound. A more thorough understanding of the prognosis could lead to more precise patient selection in the future.

Therefore, the aim of this study was to evaluate the differences in long-term prognosis and morbidity between three different groups of patients with metastatic colorectal cancer: those with peritoneal and liver metastases treated with concomitant CRS, HIPEC, and liver resection; those with peritoneal metastases only treated with CRS and HIPEC; and those with liver metastases only treated with liver resection.

## Methods

### Study Design and Population

This cohort study included all patients who underwent LRx and/or CRSH for colorectal cancer due to liver and/or peritoneal metastases at Uppsala University Hospital, a tertiary referral center, from January 2006–December 2016.

The study was approved by the Swedish Ethical Review Authority in Uppsala (Dnr. 2013/203 and 2018/086).

### Data Sources and Collection

All patients who underwent surgery with CRSH or LRx were identified from hospital-specific data registries. The inclusion criteria for the study were colorectal cancer and the initial surgery for metastatic disease (peritoneal and/or liver). The exclusion criteria were appendiceal cancer, having undergone CRSH although Peritoneal Cancer Index (PCI) [9] was 0 (thus excluding patients who underwent prophylactic HIPEC), or a Completeness of Cytoreduction Score (CCS) [9] of 2–3 (occurred in a few selected patients in the earlier part of the study period prior to more stringent adherence to standard practice).

Patient records were studied for information about BMI, age, surgery for the primary tumor and previous metastatic surgery, prior surgical score (PSS) [10], postoperative complications according to the Clavien–Dindo Classification [11] and for patients having undergone surgery for peritoneal metastases, also for information regarding preoperative and adjuvant (postoperative) chemotherapy. Adjuvant chemotherapy treatment was defined according to data regarding intention to treat. Obesity was defined as a body mass index (BMI) > 30. Metastases were defined as synchronous if they were diagnosed within 6 months of diagnosis of the primary tumor.

Operation records were studied for the location of the primary tumor [right-sided colon cancer was defined as located in the ascending or transverse colon (midgut origin) and left-sided colon cancer as located in the descending colon or the rectum (hindgut origin)], extent of peritoneal tumor involvement through PCI, completeness of CRS with CCS, intraperitoneal chemotherapy, and extent of liver resection, as applicable. For statistical purposes, the PCI was considered a baseline variable because although a definitive assessment is performed peroperatively, at the beginning of the surgery (estimations are made preoperatively), it constitutes a prominent part of the decision whether to perform CRS as planned or not. Similarly, liver tumor burden was re-evaluated with perioperative ultrasound examination before definitive resection was performed. Pathology reports were studied for information on the type of cancer. Information on the number and size of liver metastases [to calculate the Tumor Burden Score [12] (TBS)] was extracted from preoperative radiology reports (computed tomography and/or magnetic resonance imaging of the liver) and/or postoperative pathology reports. The patient data was prospectively recorded in local registries and completed as necessary through additional revision of patient records. Overall survival was calculated as the number of months from the surgical procedure to the date of death, as extracted from the Swedish Population Register. For patients having undergone surgery for peritoneal metastases date of recurrence was obtained through questionnaires to referring hospitals and calculated as above.

### Operative Methods

In patients with peritoneal metastases, the operability and PCI were evaluated after midline laparotomy. CRS was performed, with resection of involved organs and peritonectomy of affected parts of the peritoneum, according to standard techniques [13]. In patients with liver metastases, liver resection, ranging from resection of superficial tumor deposits to atypical liver resection to segmentectomies and to hemihepatectomies was performed. Peroperative liver ablation techniques were used in selected patients as a complement to resection in the liver resection only group.

### Intraperitoneal Chemotherapy

For patients with peritoneal metastases, CRS was followed by HIPEC according to the Coliseum method [14]. An inlet catheter was placed centrally in the abdomen, and four outlet catheters were placed laterally in the abdominal wall. Then, a plastic film was secured over the open laparotomy to create a closed system. The catheters were connected to a perfusion pump and temperature regulator that perfused the heated chemotherapy solution. Intraabdominal temperature was monitored with temperature probes, and maintained at 41–42 °C. The patients’ body temperature was lowered with cooling blankets and kept at 35–36 °C. HIPEC was administered using either oxaliplatin (460 mg/m^2^) or oxaliplatin and irinotecan (360–400 mg/m^2^ for both drugs) for 30 min. Peroperatively, 40 min before HIPEC treatment, intravenous 5-fluorouracil 400 mg/m^2^ and calcium folinate 60 mg/m^2^ were administered.

### Statistical Analysis

Patient characteristics are presented as medians, with the first and third quartiles, or proportions, as appropriate. Differences in medians and proportions were estimated by the Mann‒Whitney U test and the chi-square test, respectively, and adjusted for multiple comparisons using conservative Bonferroni correction.

Overall survival was displayed by the Kaplan‒Meier survival curves and evaluated for differences by the log-rank test. Univariate and multivariate Cox proportional hazard regression was used to evaluate the associations of baseline characteristics with survival using stepwise backward variable selection for variables from univariable analysis with *p* < 0.01. A crosstabulation was used to demonstrate the results for subgroups according to TBS and PCI. Statistical significance was defined as two-tailed *p* < 0.05. SPSS 28.0 software was used for all statistical analyses except for analyses regarding recurrence-free survival and chemotherapy where R studio 2023.09.1 + 494 was used.

## Results

**Fig. 1:**
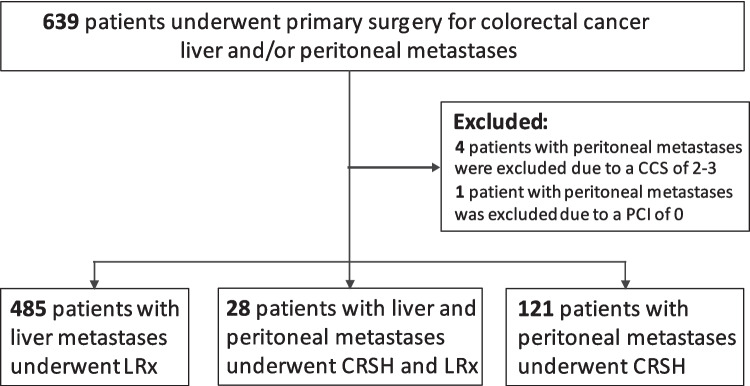
Consort diagram. Consort diagram for the 639 patients who underwent primary surgery for colorectal cancer metastases in the peritoneum and/or liver. LRx, liver resection; CRSH, cytoreductive surgery and hyperthermic intraperitoneal chemotherapy; CRSH + LRx, concomitant CRSH and LRx; CCS, Completeness of Cytoreduction Score; PCI, Peritoneal Cancer Index

A total of 639 patients underwent surgery for colorectal metastases in the liver and/or peritoneum. Five patients with peritoneal metastases only were excluded, four due to a CCS of 2–3 and one because of receiving HIPEC despite a PCI of 0 (in the setting of multiple lymph node metastases), leaving a total of 634 patients available for the analyses. Of these, 28 patients had CRSH + LRx, 121 patients had CRSH only, and 485 patients were treated with LRx only (Fig. 1).

The median follow-up time was 48.9 months (IQR 22.2–82.0); for the LRx group, it was 52.9 (IQR 24.9–90.8); for the CRSH + LRx group, it was 25.2 months (IQR 12.5–49.6); and for the CRSH group, it was 34.5 months (IQR 19.4–68.4). Thus, the follow-up time was longer in the LRx group than in both the CRSH + LRx and CRSH groups (*p* < 0.001).

### Perioperative Characteristics

There were more males in the LRx group than in the CRSH and CRSH + LRx groups, and patients who underwent CRSH only and CRSH + LRx were younger than patients who underwent LRx (Table 1). Furthermore, right-sided colon cancer was more common among patients in the CRSH + LRx and CRSH groups than in the LRx group (Table 1).

**Table 1:** Patient characteristics and perioperative data for patients who underwent primary surgery for colorectal cancer metastases in the peritoneum and/or liver

Variable	Total		LRx		CRSH + LRx		CRSH		*p*-value
	Median [Q1–Q3] or count (% of total)		Median [Q1–Q3] or count (% of total)		Median [Q1–Q3] or count (% of total)		Median [Q1–Q3] or count (% of total)		
Number of patients/group	634	(100%)	485	(77%)	28	(4%)	121	(19%)	
Sex									
Male	389	(61%)	320	(66%)	13	(46%)	56	(46%)	< 0.001*
Female	245	(39%)	165	(34%)	15	(54%)	65	(54%)	
Age	65.5	[57.7–71.1]	66.7	[59.3–72.5]	63.2	[53.6–65.3]	62.0	[48.8–68.4]	< 0.001″*
Age ≥ 70	190	(30%)	166	(34%)	1	(4%)	23	(19%)	< 0.001″*
BMI	25.7	[23.5–28.7]	25.7	[23.6–28.6]	25.2	[23.3–28.3]	26.0	[23.2–29.4]	0.866
Obese (BMI ≥ 30)	111	(18%)	83	(17%)	4	(14%)	24	(20%)	0.702″*
Primary tumor									
Colon	415	(66%)	280	(58%)	26	(93%)	109	(90%)	< 0.001″*
Rectum	214	(34%)	201	(41%)	1	(3.5%)	12	(10%)	
Both	5	(1%)	4	(1%)	1	(3.5%)	0	(0%)	
Embryonal origin									
Midgut	177	(28%)	90	(19%)	17	(61%)	70	(58%)	< 0.001″*
Hindgut	455	(72%)	393	(81%)	11	(39%)	51	(42%)	
Synchronous liver metastases	305	(48%)	296	(61%)	9	(32%)	0	(0%)	< 0.001°
Synchronous peritoneal metastases	82	(13%)	0	(0%)	17	(61%)	65	(60%)	< 0.001″*
Preoperative chemotherapy	na		na		21	(75%)	67 of 118†	(57%)	0.120
Adjuvant chemotherapy	na		na		16	(57%)	48 of 111†	(43%)	0.269
TBS	3.2	[1.6–5.4]	4.0	[2.7–6.0]	2.3	[1.4–3.9]	0	[0–0]	< 0.001°
No liver metastases	121	(19%)	0	(0%)	0	(0%)	121	(100%)	< 0.001″*
Below 3	184	(29%)	167	(34%)	17	(61%)	0	(0%)	
3 to 6	289	(46%)	278	(57%)	11	(39%)	0	(0%)	
Above 6	40	(6%)	40	(8%)	0	(0%)	0	(0%)	
PCI	0	[0–0]	0	[0–0]	15	[12–24]	12	[6–19]	< 0.001″*
No peritoneal metastases	485	(76%)	485	(100%)	0	(0%)	0	(0%)	< 0.001″*
PCI ≤ 20	113	(18%)	0	(0%)	18	(64%)	95	(79%)	
PCI > 20	36	(6%)	0	(0%)	10	(36%)	26	(22%)	
Operation time	187	[126–300]	160	[112–208]	538	[446–653]	451	[373–547]	< 0.001″*
Operation time ≥ median	314	(50%)	168	(35%)	28	(100%)	118	(98%)	< 0.001″*
Blood loss (ml)	800	[400–1500]	800	[500–1600]	1300	[500–1800]	400	[200–1000]	< 0.001″*
Blood loss ≥ median	321	(51%)	260	(54%)	19	(68%)	42	(35%)	< 0.001″*
Dindo-Clavien > 3a	76	(12%)	55	(11%)	3	(11%)	18	(15%)	0.551
30d mortality	5	(0.8%)	3	(0.6%)	1	(3.6%)	1	(0.8%)	0.228
90d mortality	14	(2.2%)	11	(2.3%)	2	(7.1%)	1	(0.8%)	0.120

*LRx* liver resection, *CRSH* cytoreductive surgery and hyperthermic intraperitoneal chemotherapy; median value with inter quartile range [Q1–Q3] and counts with percentage (%) of total number of patients/group except when specified†; *BMI* body mass index; synchronous metastases – diagnosed < 6 months of diagnosis of primary tumor; *na* not available, *TBS* Tumor Burden Score, *PCI* Peritoneal Cancer Index, *ml* milliliters

″LRx vs.CRSH + LRx

*LRx vs. CRSH

°LRx vs. CRSH + LRx vs. CRSH

Synchronous liver metastases were less common than metachronous in the CRSH + LRx group contrary to the LRx group, whereas synchronous peritoneal metastases were almost equally common in the CRSH with or without LRx groups. Three patients in the CRSH + LRx group had very superficial liver metastases, and due to insufficient documentation, a precise TBS could not be calculated. These patients were allocated a TBS < 3, which corresponded well to the macroscopic findings and resection techniques described in their operative notes. None of the patients in the CRSH + LRx group had a TBS > 6 (Table 1). For details on the extent of liver resection, see Supplementary Table 1.

### Postoperative Course

The incidence of severe complications, classified as Clavien‒Dindo scores > 3a, did not significantly differ among the LRx, CRSH, and CRSH + LRx groups, and the total 30- and 90-day mortality rates were 0.8% and 2.2%, respectively, without statistically significant differences among the three groups (Table 1). According to the logistic regression model, neither the PCI nor the TBS groups had any effect on the prediction of severe complications or 30-day mortality, but TBS was a predictor of 90-day mortality (*p* = 0.013). The detailed Clavien–Dindo scores are described in Supplementary Table 1.

### Survival

**Fig. 2:**
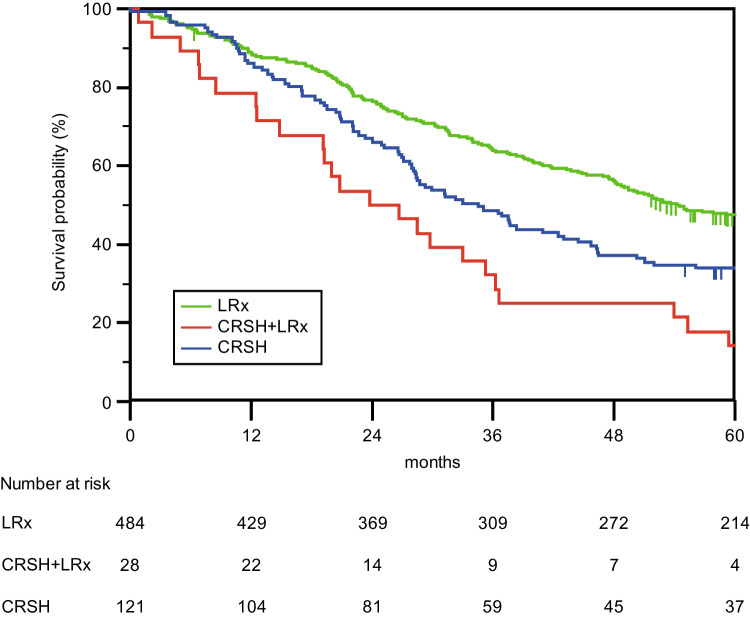
Operation Type. Overall survival for 634 patients after surgery for liver and/or peritoneal metastases with either LRx (liver resection only), CRSH + LRx (cytoreductive surgery, hyperthermic intraperitoneal chemotherapy, and liver resection), or CRSH (cytoreductive surgery and hyperthermic intraperitoneal chemotherapy only). The median survival times were 54.2 months (95% CI 47.6–61.0) for LRx, 23.8 months (95% CI 12.8–34.8) for CRSH + LRx and 34.5 months (95% CI 27.1–41.9) for CRSH. A log rank (Mantel Cox) *p*-value < 0.001 was used

The median OS in the total cohort was 49.0 months (95% CI 43.7–54.3) but was shorter for patients who underwent CRSH and liver resection or CRSH alone than for patients who underwent liver resection only (Fig. 2).

The 1-year overall survival was 88% [89% in the liver resection only group, 79% (22/28) in the CRSH and liver resection group and 86% in the CRSH only group, *p* < 0.001]. The 5-year overall survival rate was 43%. Almost half of the patients treated with liver resection only survived 5 years (47%), and approximately one in three patients treated with CRSH only (34%), whereas 14% of patients (4/28) survived 5 years when treated with CRSH and liver resection.

**Fig. 3:**
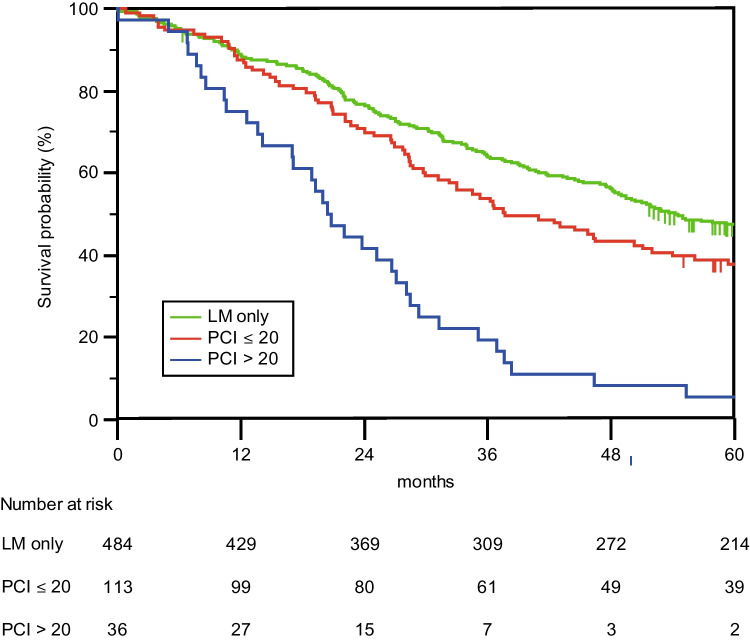
PCI. Overall survival for 634 patients after surgery for liver and/or peritoneal metastases based on the PCI (Peritoneal Cancer Index) of peritoneal metastases. LM only (liver metastases only), PCI ≤ 20 or PCI > 20). The median survival times were 54.2 months (95% CI 47.6–61.0) for LM only patients (same group as the LRx patients in Fig. 2), 37.6 months (95% CI 27.0–48.3) for patients with a PCI ≤ 20 and 20.4 months (95% CI 16.3–24.6) for patients with a PCI > 20. A log rank (Mantel Cox) *p*-value < 0.001 was used

**Fig. 4:**
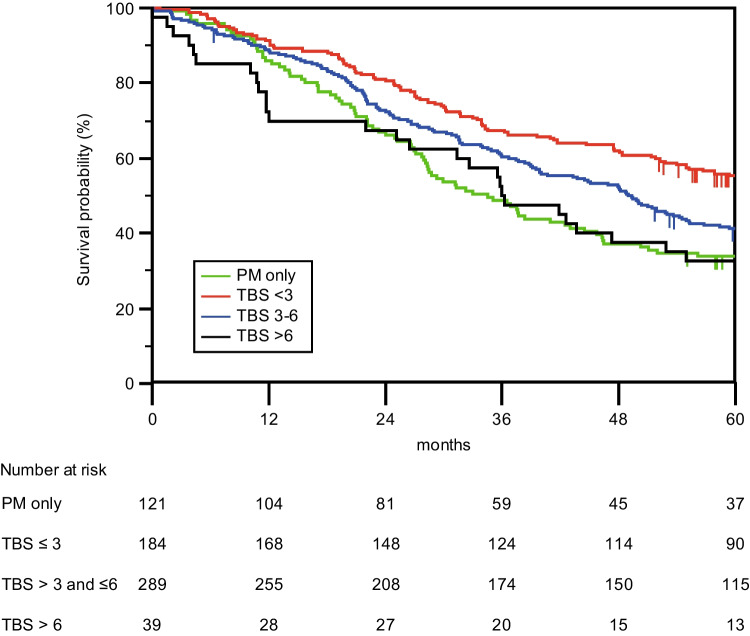
TBS. Overall survival for 634 patients after surgery for liver and/or peritoneal metastases based on the TBS (Tumor Burden Score) of liver metastases. PM only (peritoneal metastases only), TBS < 3, TBS 3–6, or TBS > 6. The median survival times were 34.5 months (95% CI 27.1–41.9) for the PM only patients (same group as the CRSH patients in Fig. 2), 66.5 months (95% CI 52.6–80.4) for patients with a TBS < 3, 49.3 months (95% CI 42.2–56.5) for patients with a TBS of 3–6 and 35.9 months (95% CI 26.2–45.7) for patients with a TBS > 6. A log rank (Mantel Cox) *p*-value < 0.001 was used

Increased PCI and TBS were associated with decreased OS (Figs. 3 and 4). Cross-tabulation analyses of TBS (0, < 3, 3–6, > 6) and PCI (0, 1–20, 21–39) groups revealed decreased survival at a PCI > 20, irrespective of the TBS (Table 2). A higher TBS and PCI were associated with an increased risk of death according to the univariate Cox proportional hazard regression (Table 3). Multivariate Cox proportional hazard regression identified high TBS, high PCI, and age > 70 years as independent predictors for increased risk of death. When the survival model was adjusted for known prognostic factors from the operation (blood loss during operation) and postoperative outcomes (severe complications), the importance of TBS and PCI increased even further (Table 3).

**Table 2:** Cross-tabulation of Tumor Burden Score and Peritoneal Cancer Index groups presenting median overall survival in months after surgery for liver and/or peritoneal metastases

	No	Liver metastases only		No	PCI ≤ 20		No	PCI > 20	
		Median (95% CI) OS (months)			Median (95% CI) OS (months)			Median (95% CI) OS (months)	
Peritoneal metastases only				95	43	(29–57)	26	22	(12–32)
TBS < 3	167	73	(51–96)	10	36	(31–42)	7	13	(0–27)
TBS 3–6	278	49	(44–55)	8	19	(0–38)	3	27	(22–31)
TBS > 6	40	36	(26–46)						

*Median OS* median overall survival in months for patients who underwent surgery for colorectal cancer metastases in the peritoneum and/or liver, *(95% CI)* 95% confidence interval; Log Rank test, *p* < 0.001; *TBS* Tumor Burden Score, *PCI* Peritoneal Cancer Index

**Table 3:** Results from univariate and multivariate cox proportional hazard regressions for risk of death for patients who underwent surgery for colorectal cancer metastases in the peritoneum and/or liver

	Univariate Cox proportional-hazard regression			Multivariate Cox proportional-hazard regression—preoperative variables only			Multivariate Cox proportional-hazard regression—pre- and per-operative variables only		
	HR	95% CI	*p*-value	HR	95% CI	*p*-value	HR	95% CI	*p*-value
Sex—male	1.051	(0.866–1.276)	0.615						
Age ≥ 70 y	1.189	(0.971–1.457)	0.094	1.376	(1.114–1.699)	0.003	1.388	(1.123–1.716)	0.002
BMI > 30	1.050	(0.823–1.339)	0.696						
Primary tumor location									
Rectum			0.441						
Colon	1.036	(0.849–1.265)	0.726						
Both	0.428	(0.106–1.729)	0.233						
Embryonal origin									
Hindgut									
Midgut	1.067	(0.866–1.314)	0.543						
Synchronous liver metastases	0.886	(0.734–1.069)	0.206						
Synchronous peritoneal metastases	1.523	(1.165–1.992)	0.002						
TBS									
No liver metastases			0.001						
Below 3	0.613	(0.463—0.812)	0.001	1.522	(0.946–2.449)	0.083	1.547	(0.965–2.482)	0.070
3 to 6	0.823	(0.642–1.056)	0.125	2.116	(1.314–3.407)	0.002	2.030	(1.253–3.290)	0.004
Above 6	1.132	(0.759–1.688)	0.542	3.216	(1.780–5.809)	< 0.001	3.148	(1.735–5.712)	< 0.001
PCI									
No peritoneal metastases			< 0.001						
PCI ≤ 20	1.245	(0.974–1.591)	0.080	2.400	(1.519–3.791)	< 0.001	2.458	(1.559–3.873)	< 0.001
PCI > 20	3.385	(2.374–4.828)	< 0.001	5.999	(3.667–9.814)	< 0.001	5.654	(3.422–9.343)	< 0.001
Blood loss above median	1.446	(1.197–1.748)	< 0.001				1.323	(1.082–1.618)	0.006
Clavien > 3a	1.547	(1.178–2.032)	0.002				1.476	(1.105–1.971)	0.008

Risk of death for patients who underwent surgery for colorectal cancer metastases in the peritneum and/or liver; *HR* hazard ratio, *95% CI* 95% confidence interval, *TBS* Tumor Burden Score, *PCI* Peritoneal Cancer Index, *LRx* liver resection, *CRSH* cytoreductive surgery and hyperthermic intraperitoneal chemotherapy

**Fig. 5:**
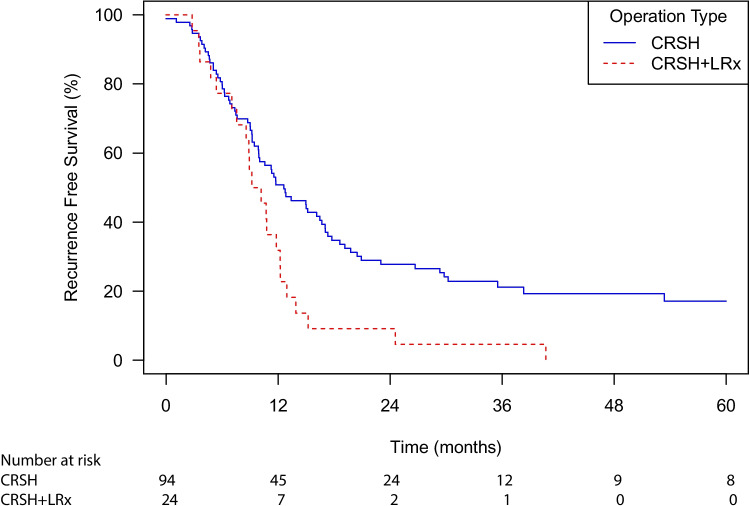
Recurrence free survival after CRSH and CRSH + LRx. Recurrence free survival for 118 of 149 patients (15 patients were excluded due to CCS = 1 and 16 patients had missing data) after surgery for peritoneal and liver metastases only or peritoneal metastases only with either CRSH + LRx (cytoreductive surgery, hyperthermic intraperitoneal chemotherapy and liver resection), or CRSH (cytoreductive surgery and hyperthermic intraperitoneal chemotherapy only). The median survival times were 9.7 months (95% CI 8.6–12.3) for CRSH + LRx and 12.6 months (95% CI 10.0–17.0) for CRSH. A log rank (Mantel Cox) *p*-value < 0.008 was used

Total median recurrence-free survival for the CRSH and liver resection and CRSH only groups was 11.6 months (95% CI 9.9–13.9) (Fig. 5).

## Discussion

In this study, the median overall survival of patients who underwent concomitant cytoreductive surgery, HIPEC, and liver resection was less than half that of patients who underwent liver resection only, whereas survival of patients treated with cytoreductive surgery and HIPEC only fell between that of patients in the other groups. Increased TBS and PCI were associated with decreased overall survival. Severe postoperative complications with a Clavien–Dindo score > 3a were comparable among all groups.

The presented results are similar to those of studies by Elias et al. [15] and Delhorme et al. [16], where the 5-year overall survival rates for patients who underwent the same type of surgery were 26.4% and 22%, respectively, after concomitant surgery; 36.5% and 54%, respectively, after cytoreductive surgery and HIPEC; and 38.5% and 40%, respectively, after liver resection only, and not significantly different. However, in the current study, survival after liver resection only was significantly improved.

A more recent study by Pinto et al. [17] compared patients who underwent surgery for peritoneal metastases to those who underwent surgery for both peritoneal and liver metastases, but the patients in the latter group underwent either a concomitant resection of metastases or a two-step procedure. They observed overall survival times of 65 and 31 months (*p* = 0.188) respectively, which also corresponded to our results. However, Lee et al. [18] reported a significant difference in 2-year survival rates between patients who underwent concomitant surgery (62%) and patients who underwent CRS and HIPEC (79%) (*p* < 0.001) in their study, which included patients with invasive appendiceal and colorectal cancer.

According to a meta-analysis by Zou et al. [19], the pooled hazard ratio for overall survival was 1.68 (95% CI 1.33–2.13, *p* < 0.01) for patients who underwent surgery for both peritoneal and liver metastases versus for patients who underwent surgery for peritoneal metastases only. Similarly, the pooled hazard ratio for 5-year overall survival reported by Flood et al. [7] was 1.24 (95% CI 1.08–1.43, *p* = 0.002). Both meta-analyses found no significant difference between these groups in their pooled risk ratio for postoperative mortality, in agreement with the present study. However, the pooled risk ratio for major morbidity significantly favored patients with peritoneal metastases only, whereas Flood et al. [7] found no significant difference, similar to the present study.

Elias et al. [15] and Lee et al. [18] both reported that an increase in the number of liver metastases and in the PCI were negatively associated with survival. This corresponds to the presented findings as well as to the findings of Grange et al. [20], although we both used TBS [12], which, in addition to the number of liver metastases, takes the size of the largest liver metastasis into consideration.

Interestingly, in the current study, one can discern a tendency for PCI to impact survival more than TBS, illustrated by the more evident separation of survival curves according to PCI categories compared with TBS curves and the higher odds ratios in survival analyses. A possible consequence might be that a greater weight could be attributed to PCI relative to TBS in preoperative selection; therefore, patients with a greater TBS might still benefit from concomitant cytoreductive surgery, HIPEC, and liver resection in the setting of a low PCI. In a multicenter study by Lo Dico et al. [21], OS was as high as 44.8 months after concomitant surgery in a cohort of patients where 68.5% had a PCI < 12, and Grange [20] et al. reported an OS of 45 months in their cohort of patients with a PCI ≤ 12 and a TBS ≤ 3.

Although a cut-off of PCI > 20 is high and commonly known for poor survival (Goéré et al. [22]), these patients constitute part of the true patient cohort in our clinical setting where we do not have a fixed upper limit for PCI. Therefore, these patients were included in the study. Despite their high PCI, these patients were specifically selected after discussion at multidisciplinary conferences to undergo surgery based on other preferential factors, such as tumor-specific variables, response to prior therapy, time to relapse, extent and resectability of metastases, age, and comorbidities.

When considering whether concomitant surgery for peritoneal and liver metastases is an option for selected patients, it is important to appraise the possible survival after best-practice chemotherapy. In the aforementioned studies by Franko et al. [4] and van der Geest et al. [5], the median survival for patients with either peritoneal or liver metastases improved over time but was lower than that for the selected group of patients in the current study who underwent combined surgery.

As the current study was register based, it was limited by the information provided in the hospital’s patient records and registries. There was also a notable difference in the number of patients in the groups. However, at the same time, this reflects the clinical setting where only selected groups of patients are offered surgery, especially for peritoneal metastases and even more so when both liver and peritoneal metastases are present. For this study, the choice was made to include all patients who underwent surgical procedures in the setting of an adequate indication. Another limitation was the relatively long study period, which entails that both surgical and systemic treatments developed during the study period. When analyzing operation date order and time during the study, no impact on survival was observed. Nevertheless, all patients were discussed at multidisciplinary conferences by oncologists and cytoreductive and liver surgeons and treated according to national guidelines, thus including decisions on preoperative and adjuvant chemotherapy. The variables included in the study were recorded in a uniform prospective manner in local registries. As a consequence data regarding preoperative and adjuvant chemotherapy as well as recurrence-free survival was not available for the liver resection only group. Yet, according to Swedish National Guidelines [23] on treatment of colorectal liver metastases, preoperative chemotherapy is recommended in most instances but not routinely for solitary metachronous liver metastases (depending on size and location) as complete radiological response could lead to difficulties achieving successful local treatment. A study by Scherman et al. [24], which included several Swedish centers, found that 86% of patients with liver metastases who were treated with curative intent received chemotherapy, 63% then recurred but subsequently 53% of these patients was retreated with curative intent.

Hopefully, the presented results of long-term survival of patients with colorectal peritoneal and liver metastases who undergo concomitant cytoreductive surgery, HIPEC, and liver resection will improve the knowledge of the prognostic value of TBS and PCI in this setting. In the future, this might be a step to further improve patient selection and help identify patients who could benefit from this taxing and extensive surgical procedure.

## Conclusions

Patients treated with cytoreductive surgery, hyperthermic intraperitoneal chemotherapy, and liver resection for liver and peritoneal metastases experienced a 2-year long median overall survival although overall survival was shorter than for patients treated with cytoreductive surgery and hyperthermic intraperitoneal chemotherapy or liver resection only. Furthermore, the rates of severe postoperative complications were similar in these three groups. The presence and extent of peritoneal spread seem to impact survival more than the tumor burden in the liver. Therefore, colorectal liver metastases cannot be considered an absolute contraindication for cytoreductive surgery, hyperthermic intraperitoneal chemotherapy, and liver resection in patients with concomitant peritoneal metastases. Meticulous patient selection enables the benefits of this aggressive oncosurgical approach without increasing postoperative morbidity.

## Supplementary Information

Below is the link to the electronic supplementary material.

Supplementary file1 (PDF 109 KB)

## Data Availability

All trial data is stored in Uppsala University’s storage system for research data, Dataportal Allvis, and may be provided by the corresponding author upon request.

## Abbreviations

LRx: Liver resection
CRS: Cytoreductive surgery
HIPEC: Hyperthermic intraperitoneal chemotherapy
CRSH: CRS and HIPEC
CRSH + LRx: Combination procedure of CRS, HIPEC, and LRx
TBS: Tumor Burden Score
PCI: Peritoneal Cancer Index
PSS: Prior surgical score
CCS: Completeness of Cytoreduction Score
OS: Overall survival
CI: Confidence interval
