# Supplementary material for: The Extent of Tumor in the Peritoneum and Liver Influences Outcomes After Surgery for Synchronous Liver and Peritoneal Colorectal Metastases: A Cohort Study
Source: J Gastrointest Cancer. 2024 Nov 14;56(1):19. doi: 10.1007/s12029-024-01139-y (PMC11561098; doi:10.1007/s12029-024-01139-y)
Supplement: Supplementary file 1 — Supplementary file1 (PDF 109 KB) [file 12029_2024_1139_MOESM1_ESM.pdf]

The Extent of Tumor in the Peritoneum and Liver Influences Outcomes after Surgery for Synchronous Liver and Peritoneal Colorectal Metastases: a Cohort Study

Journal of Gastrointestinal Cancer

Pearl Sanchez Salas, Jozef Urdzik, Wilhelm Graf, Bengt Isaksson, Helgi Birgisson

## Supplementary Table 1

Additional Peroperative and Postoperative data for patients who underwent primary surgery for colorectal cancer metastases in the peritoneum and/or liver regarding extent of liver resection and postoperative complications.

| variable | | LRx count (% of total) | | CRSH+LRx count (% of total) | | CRSH count (% of total) | | p-value |
|---|---|---|---|---|---|---|---|---|
| Number of patients / group | | 485 | (77 %) | 28 | (4 %) | 121 | (19 %) | |
| Liver resection - | wedge | 97 | (20 %) | 20 | (71 %) | | | < 0.001" |
| | up to 2 segments | 132 | (27 %) | 6 | (21 %) | | | |
| | major | 220 | (45 %) | 2 | (7 %) | | | |
| | extended | 36 | (7 %) | 0 | (0 %) | | | |
| Clavien-Dindo - | 0 | 198 | (41 %) | 0 | (0 %) | 0 | (0 %) | < 0.001"* |
| | 1 | 58 | (12 %) | 6 | (21 %) | 22 | (18 %) | |
| | 2 | 96 | (20 %) | 15 | (54 %) | 67 | (55 %) | |
| | 3a | 78 | (16 %) | 4 | (14 %) | 14 | (12 %) | |
| | 3b | 21 | (4 %) | 2 | (7 %) | 8 | (7 %) | |
| | 4a | 14 | (3 %) | 1 | (4 %) | 8 | (7 %) | |
| | 4b | 16 | (3 %) | 0 | (0 %) | 1 | (1 %) | |
| | 5 | 4 | (0.8 %) | 0 | (0 %) | 1 | (0.8 %) | |

Additional data regarding extent of liver resection and postoperative complications for patients who underwent surgery for colorectal cancer metastases in the liver and/or peritoneum; LRx - Liver Resection; CRSH - Cytoreductive Surgery and Hyperthermic Intraperitoneal Chemotherapy; Counts with percentage (%); " - LRx vs.CRSH+LRx ; * - LRx vs. CRSH ; ° - LRx vs. CRSH+LRx vs. CRSH

Corresponding author: Pearl Sanchez Salas
Department of Surgical Sciences, Uppsala University
e-mail: pearl.sanchez-salas@uu.se
